# Managing Oncology Services During a Major Coronavirus Outbreak: Lessons From the Saudi Arabia Experience

**DOI:** 10.1200/GO.20.00063

**Published:** 2020-03-27

**Authors:** Abdul-Rahman Jazieh, Abdulrahman Al Hadab, Ashwaq Al Olayan, Ayman AlHejazi, Faisal Al Safi, Abullah Al Qarni, Faisal Farooqui, Nashmia Al Mutairi, Thamer H. Alenazi

**Affiliations:** ^1^ Department of Oncology, King Abdulaziz Medical City, King Saud bin Abdulaziz University for Health Sciences, King Abdullah International Medical Research Center, Riyadh, Saudi Arabia; ^2^ Infection Disease Division, Department of Medicine, King Abdulaziz Medical City, King Saud bin Abdulaziz University for Health Sciences, King Abdullah International Medical Research Center, Riyadh, Saudi Arabia

## Abstract

Outbreaks of infectious etiology, particularly those caused by a novel virus that has no known treatment or vaccine, may result in the interruption of medical care provided to patients with cancer and put them at risk for undertreatment in addition to the risk of being exposed to infection, a life-threatening event among patients with cancer. This article describes the approach used to manage patients with cancer during a large-scale Middle East respiratory syndrome–coronavirus hospital outbreak in Saudi Arabia to ensure continuity of care and minimize harm from treatment interruption or acquiring infection. The approach taken toward managing this high-risk situation (COVID-19) could be easily adopted by health care organizations and would be helpful to ensure readiness for the occurrence of future outbreaks of different infectious etiologies like those recent episodes of new coronavirus.

## INTRODUCTION

The recurrent outbreaks of coronaviruses in different parts of the world pose a major challenge to health care systems and expose patients and staff to serious risk. These outbreaks may lead to an interruption in health care services and the provision of care for patients, posing additional risks to them.

These outbreaks pose a greater threat to patients with a chronic disease in terms of morbidity and mortality compared with healthy individuals due to the increased vulnerability of the patient secondary to their disease.^1,2^ Patients with cancer are immunocompromised and therefore more vulnerable to infection, which can often end in fatality. The vulnerability of patients with cancer was evident by the high risk of mortality during the previous Middle East respiratory syndrome–coronavirus (MERS-CoV) outbreaks (see the article by Jazieh et al^3^ in this issue). Besides the risk of infection for exposed patients, additional major risks occur because of the interruption of the health care services provided at these facilities when they are subsequently shut down.

Cancer is a life-threatening disease that should be treated in a timely fashion to avoid a detrimental outcome to patients and minimize their physical and emotional suffering. The provision of cancer care is complex and requires multiple teams of professionals with specialized expertise and access to sophisticated, expensive resources. The complexity of cancer care already presents major challenges to the health care systems in affluent countries; national reports from both the United States and United Kingdom identified the crisis situation already faced in the provision of this care.^4-6^ This is the situation internationally in normal daily practice without external complicating factors, such as war or infectious outbreaks. One can imagine the challenges that arise in developing countries while facing such external crises, which present an additional burden to inherently complex care.^7,8^

MERS-CoV was the cause of multiple outbreaks in Saudi Arabia, which spread internationally.^9-11^ The outbreak of MERS-CoV in June 2015 led to the closure of our hospital, as well as other hospitals within the city of Riyadh.^12^ This crisis posed a serious challenge to our staff and patients. The Oncology Department, in particular, faced unique challenges because of many factors, including infected oncology patients, a large susceptible patient population, interruption of services provided to our patients, and additional risks to our staff.

With recent outbreaks of the new coronavirus in China and other countries, the factors related to oncology patients’ care and corresponding outcomes are a major concern for the oncology community; therefore, this article describes the approach used to manage oncology services in response to the MERS-CoV outbreak and the implications of hospital closure. The real-life experiences and subsequent recommendations may provide some guidance and a supportive framework for oncologists to use in affected areas.

**Context**

**Key Objective**
With recurrent infection outbreaks from different pathogens, patients with cancer are at high risk for harm because of the susceptibility to infections and the interruption of care, in addition to the associated risk of underlying cancer and its treatment.
**Knowledge Generated**
In response to the 2015 coronavirus outbreak in our country, we developed a detailed plan to help manage oncology services to prevent harm to our patients or staff. The plan focused on managing infected patients, preventing any new infections in patients or staff, ensuring continuity of cancer care, and incorporating measures to sustain these interventions far into the postoutbreak period.
**Relevance**
The described plan may serve as a platform to manage oncology services during similar crises that pose serious risks for patients and staff.

### The Crisis Management Plan

In coordination with the organizational leadership, the oncology service leaders developed a plan to manage the crisis with 3 main objectives: to treat affected patients, prevent further infection to patients and staff, and deliver timely, safe cancer care for all patients. The Crisis Management Plan included 5 main components: leadership and communication, patient management, staff management, infection control, and recovery plan (Table 1). This plan presents a comprehensive approach to control the harm that resulted from the outbreak, whether from infection or interruption of treatment.

**TABLE 1:**
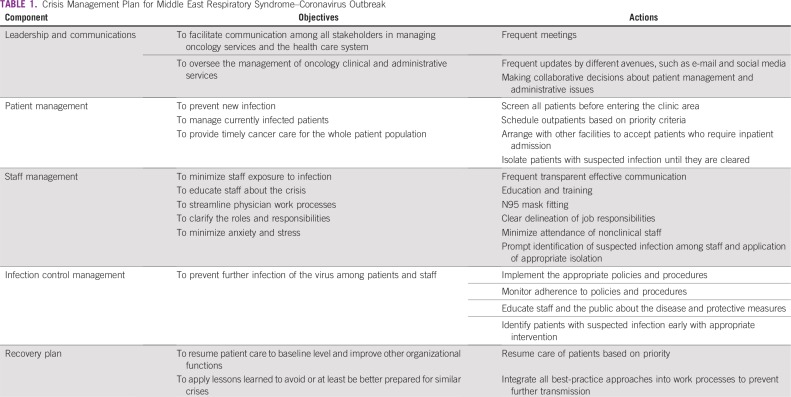
Crisis Management Plan for Middle East Respiratory Syndrome–Coronavirus Outbreak

#### Leadership and communication plan.

To be effective, leadership must be engaged and visible, and timely communication is imperative for any organization’s continuing functionality. These 2 issues are even more relevant during a crisis, making them paramount for crisis management.

A leadership committee was formed to manage the oncology services during the crisis; this included the Chairman, Deputy Chairman, Section Heads, Operations Administrator, Nursing Manager, and Quality Improvement Specialist. The main objectives of this committee were to communicate to the oncology department staff the information received from the daily Hospital Command Center meetings, discuss the current status of the outbreak in the whole hospital, and assess the risk for oncology patients. The team discussed the current admitted patients under the oncology service and their management plan, and proposed a contingency plan for their management, if needed. The group met as a whole or as subgroup at least twice a week and discussed any updates related to the outbreak, any changes in patient care, challenges, and the communication plan.

The leadership frequently corresponded by using social media tools, such as WhatsApp and organizational e-mails. The communication with the remaining departmental staff was performed in person through meetings with the Section Heads or through departmental e-mails to all staff regarding situational updates and organizational expectations.

**FIG 1:**
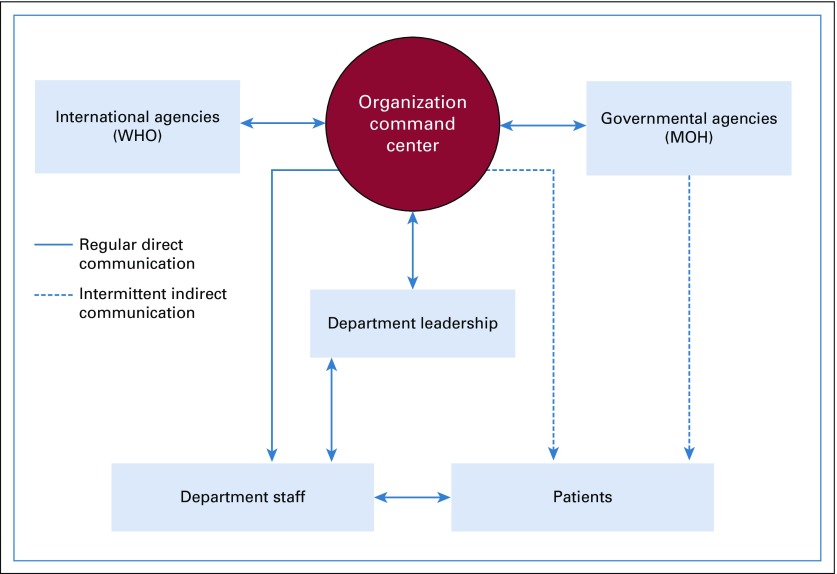
Department staff received a direct message from the organization leadership and Department leadership committee. The Hospital leadership received communication from WHO, the Ministry of Health (MOH), the Department leadership, staff, and patients. Patients received direct communication from Department staff and general communication from organization leadership (Command Center) and MOH.

Staff were encouraged to review the MERS-CoV page within the organization Web site and to be aware of the Command Center correspondence by e-mail disseminated on a daily basis initially to all staff. Of note, these communications were bidirectional to ensure better awareness of all aspects of the crisis in a timely fashion (Fig 1).

#### Patient management.

The patient management plan had 3 main aims: to prevent new infection in the oncology service, to manage currently infected patients, and to provide timely cancer care for the whole patient population. Specific plans were developed based on the setting, outpatient services, and inpatient services.

### Outpatient Setting

- Early in the outbreak, all the appointments were canceled for 4 days to obtain a better understanding of the situation and to develop an appropriate action plan.
- Medical records of all scheduled patients were reviewed by the primary oncologist, and patients were divided into 3 categories.
  - Urgent (need to be seen on time): patients who were scheduled for chemotherapy that could not be postponed or were due for disease assessment; we kept the scheduled appointments for these patients.
  - Intermediate: patients who could be rescheduled after a short delay (up to 2-4 weeks); the appointments for these patients were postponed.
  - Routine follow-up: appointments were rescheduled as far in the future as deemed clinically safe for the patient without any foreseeable negative impact.
- All patients were screened before entering the clinic with a checklist that included clinical and epidemiologic criteria; anyone with a suspected infection was referred to a triage area segregated from the general population.
- The infusion unit working hours were extended from 8:00 am to 8:00 pm to accommodate any delayed patients. Medical staff coverage was secured onsite, and the working hours of the satellite pharmacy were extended.
- Walk-in patients: Patients who showed up to the clinic without an appointment were screened for infection and were either directed to the triage area for suspected infection or seen by physicians covering the clinic, according to the result of the screening checklist.
- Oncology physicians managed patients who could be seen in the outpatient setting. Patients who required admission were sent to an alternative hospital. Patients were transported via hospital ambulance if their condition was unstable; patients in stable condition were transferred by private car. All referred patients were accompanied by a completed Transfer Form and written medical summary report of their treatment with pertinent data.

### Inpatient Care for Admitted Patients

**FIG 2:**
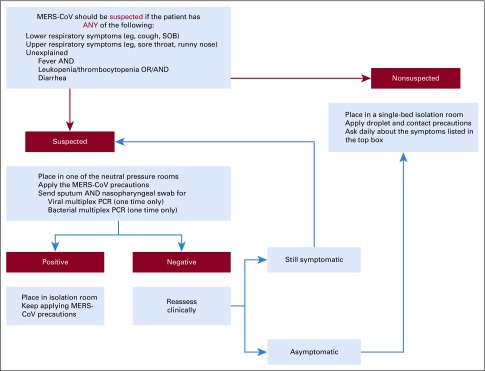
Middle East respiratory syndrome–coronavirus (MERS-CoV) algorithm for admitted oncology patients. PCR, polymerase chain reaction; SOB, shortness of breath.

The inpatient management plan was designed with Infection Control and Nursing.

a. Every patient’s condition was reviewed, and all stable patients were discharged, if possible. The census was reduced from 79 patients on August 19, 2015, to 23 patients on September 10, 2015.
 b. The patients who were not candidates for discharge remained in the hospital with close monitoring, and if any patient met the definition for suspected infection, she or he was placed on airborne isolation precautions (Fig 2).

### New Admission Protocol

The organization reached an agreement to use a certain number of beds within an alternative hospital to enable the admission of our patients. A team of physicians and coordinators was assigned to cover the patients we admitted to that hospital. Timely information and support were provided to physicians in the other hospitals who were taking care of our oncology patients admitted to their services. This included a detailed treatment plan, updated medical reports, and chemotherapy order protocols.

#### Staff management.

The aim of the staff management plan was to minimize staff exposure to infection, educate them about the crisis, streamline their work process by clarifying their roles and responsibilities, and minimize their anxiety and stress. Minimizing the risk of staff exposure included making sure that all the staff underwent N95 mask fit testing, scheduling all the department’s staff to attend the hospitalwide educational training on donning and doffing clothing, applying strict hand hygiene, and implementing infection prevention precautions. All staff (physicians and nurses) were familiarized with the case definition of suspected MERS-CoV infection.

For the clinical staff, we clarified the essential tasks to manage patients, such as reviewing patients’ records, prioritizing their care, and providing timely care. All staff were instructed and educated about proper hand hygiene. Nonclinical staff were released from duty for a few days to make sure that they were not exposed to the infection, and on their return to work, minimal interaction with clinical staff was advised. All staff with respiratory symptoms were clearly instructed not to report to work; they were sent for further assessment and instructed not to have contact with other health care professionals.

#### Infection control management.

Managing infection control during an outbreak is aimed at stopping the spread of the infection among the patients and staff by ensuring implementation of appropriate policies and procedures. Dissemination of the correct knowledge and a thorough understanding of the transmission mechanism and protective measures were paramount to ensure that clinical practice was based on evidence, not myth and rumor.

The plan for infection control included:

 a. Timely information was communicated from the leadership committee to the staff and all affected services via e-mail or in face-to-face meetings.
b. Patient management included screening all patients coming to the outpatient clinic, screening all visitors before entering the oncology wards, and implementation of the MERS-CoV management plan for inpatient units.
c. Staff and visitors were screened before entering the oncology wards with a sign-in log. Sick staff were kept away from the hospital. All staff were fitted for N95 masks, with training for all staff related to personal protective equipment. All staff were screened by nasal swab and MERS-CoV polymerase chain reaction testing to identify asymptomatic carriers; this was to ensure exclusion from the workplace and to enforce home isolation if a positive result was obtained.

#### Recovery plan.

After assurance that the outbreak was under control, we implemented the recovery plan in phases, with the goal of resuming patient care at our facility based on clinical priority. The recovery phases were:

- Phase 1: Immediate phase, 0-4 weeks. Resumed the critical services that could not be performed in the alternative hospitals, such as certain specialized surgeries, chemotherapy administration, stem cell transplantation, or radiotherapy application. Observed all the precautions for the control of infection.
- Phase 2: Intermediate phase, 5-16 weeks. Resumed all services provided before the crisis.
- Phase 3: Long-term phase: Strategic transformation of the department to provide better and safer care, integrating all new processes into the workflow to avoid newer outbreaks or in-hospital transmission.

### Measuring the Impact

The described plan resulted in the prevention of any new infection, manifested by zero cases of in-hospital transmission of MERS-CoV infection among oncology patients, not just during the months of the crisis, but over the next 4 years (the time of this report). There was a clear transformation of many work processes and departmental functions, especially among those focusing on preventing Emergency Department overcrowding, which was identified as the primary cause of the outbreak.

### Take-Home Message

The described plan was effective in the prevention of further harm to our patients from this deadly virus, a benefit that extended way beyond the outbreak. There are many lessons that can be applied to a similar crisis while taking into account that each health care facility has its own particular circumstances. The application of what we learned and the processes we implemented may vary depending on the health care facility’s specifications related to the type of patients served, the facility size and services, the health care system in the locality, and the community at large. Collaboration among different health care facilities and providers is critical, including exploring resources available outside the walls of the facility.

The key lesson that we learned in managing such outbreaks was the importance of effective communication with all stakeholders and addressing everyone’s concerns, while bearing in mind the best interests of our vulnerable patient population. We learned that it is important to have a robust mechanism to prioritize patients to ensure the provision of timely care while preventing further harm by guiding staff to provide care while staying safe.

Sustaining the improvement in the work process and flow is paramount; this cannot be achieved without support from the organization’s top leaders and stakeholders and the commitment and dedication of the staff, especially toward interventions that prevent the causes of the outbreak, such as Emergency Department overcrowding.
